# The Double-Protonation of Dihapto-Coordinated Benzene Complexes: An Enabling Strategy for Dearomatization Using Aromatic Nucleophiles

**DOI:** 10.21203/rs.3.rs-2409637/v1

**Published:** 2023-02-01

**Authors:** Jacob Smith, Justin Weatherford-Pratt, Jeremy Bloch, Megan Ericson, Jeffrey Myers, Karl Westendorff, Diane Dickie, Walter Harman

**Affiliations:** University of Virginia

## Abstract

Friedel Crafts Arylation (the Scholl reaction) is the coupling of two aromatic rings with the aid of a strong Lewis or Bronsted acid. This historically significant C-C bond forming reaction normally leads to aromatic products, often as oligomeric mixtures, dictated by the large stabilization gained upon their rearomatization. In this preliminary communication, we demonstrate how the pre-coordination of benzene by tungsten disrupts the natural course of this reaction sequence, allowing for Friedel-Crafts Arylation *without rearomatization or oligomerization*. Subsequent addition of a nucleophile to the coupled intermediate leads to functionalized cyclohexenes. The key feature of this reaction is a tungsten complex bound through two carbons, which enables a rarely observed *double protonation* of the bound benzene, and subsequent coupling to the second arene without the need of precious metal or Lewis acid catalysts.

## Introduction

**Figure 1:**
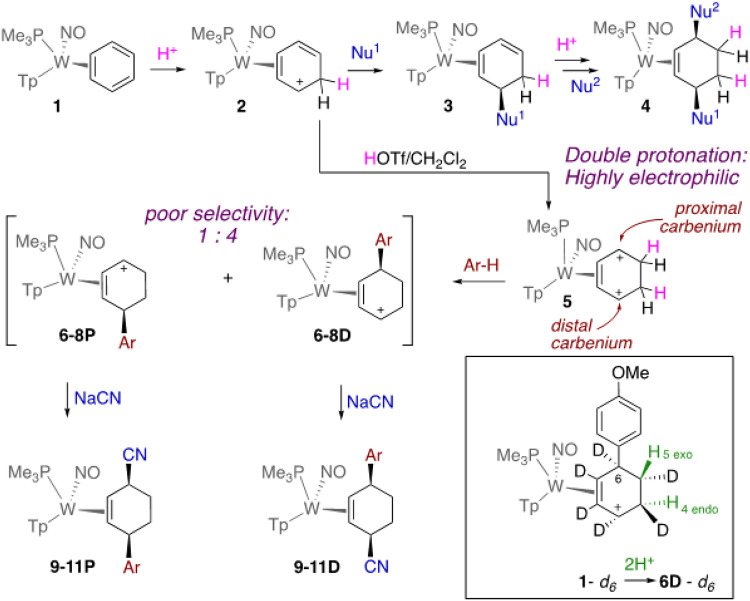
The tungsten-promoted double-protonation of benzene followed by electrophilic aromatic substitution. **6**, **9**: Ar-H = anisole; **7**, **10**: Ar-H = phenol; **8**, **11**: Ar-H = thiophene. Yields: **6-8**: 25-64%; **9**-**11**: 50-70%.

Whereas organic alkenes are widely known to undergo a reaction sequence of protonation followed by nucleophilic addition (i.e., *alkene electrophilic addition*), such a process is generally not accessible to benzenes, owing to the highly stabilized aromatic ring. However, we recently demonstrated that this reaction sequence was not only possible for a dihapto-coordinated benzene complex (Figure 1),^1, 2^ but that the resulting h^2^-diene complex (**3**) could participate in a second protonation/nucleophilic addition sequence to form the corresponding *cis*-3,6-disubstituted cyclohexene complex (**4**).^2^ The range of nucleophiles that can be added in this manner includes cyanide, enolates, Grignard reagents, amines, and alkoxides.^2^ However, the highly π-basic nature of the tungsten system required to coordinate and activate the benzene ring also stabilizes the h^2^-arenium intermediate (**2**), thereby diminishing its ability to react with so-called "π-nucleophiles" such as arenes and alkenes.^3^ We posited that a different strategy, in which the benzene was first *double-protonated*, might be possible. The resulting dicationic species (**5**), if accessible, would be highly electrophilic, and should be capable of reacting with much milder nucleophiles than its monoprotonated precursor **2**. The resulting π-allyl species (e.g., **6**-**8**) would then be positioned to react with a second nucleophile to generate 3,6-disubstitued cyclohexene complexes (e.g., **9-11**).

## Results And Discussion

We initially focused on the parent benzene complex, WTp(NO)(PMe_3_)(η^2^-benzene) (**1**), which we have previously shown could be protonated by a diphenylammonium salt (pK_a_ ~1) to generate the η^2^-arenium complex **2** (Fig. 1).^1^ When the η^2^-arenium species **2** is treated with a CD_2_Cl_2_ solution of triflic acid (HOTf; T = 0°C), the ^1^H NMR spectrum reveals that a second protonation occurs on the benzene ring to form **5**: NOESY and HSQC NMR data indicate two adjacent diastereotopic methylene groups (Fig. 1). Repeating the reaction in neat DOTf at −60°C and gradually warming the solution to 0°C indicates that the initial reaction of **2** and acid generates a paramagnetic complex as indicated by three broad peaks from 7–8.5 ppm. These signals gradually give way to the doubly protonated complex **5** - **d_2_** as the brown solution turns deep orange. In contrast, if the benzene complex **1** is subjected to HOTf in CD_2_Cl_2_ at −30 C, only the monoprotonated complex **2** is formed; this solution evolves to form **5** only after warming to 0°C. Attempts to isolate the dicationic complex **5** by precipitation with ether resulted in decomposition. However, when **5** was generated *in situ* and treated with anisole, phenol, or thiophene at −30°C, an electrophilic aromatic substitution (EAS) reaction occurred between the free aromatic and the "carbenium" of **5** proximal to the PMe_3_. Addition of the arene occurred anti to the metal to form η^2^-allyl complexes **6–8D**. This reactivity significantly differs from the precursor η^2^-benzenium complex **2**, which shows no signs of reactivity with aromatic compounds, save for indole.^2^ The resulting η^2^-allyl species (also referred to as hyperdistorted η^3^-allyl, ^4^ or σ-π distorted^5-7^), are heavily weighted toward the conformer with the carbenium carbon distal to the PMe_3_.^4^ Subsequently, the addition of a second nucleophile (CN^−^) ^2, 4^ resulted in *cis*-3,6-disubstituted cyclohexene complexes **9–11D**. Unfortunately, these products were all accompanied by roughly 20% of a second isomer, both for the η^2^-allyl intermediate (**6–8P**) and for the final cyclohexene complex (**9–11P**). These minor products were ultimately characterized (*vide infra*) as diastereomers of the major cyclohexene products in which the free arene added to the carbenium distal to the PMe_3_, and the second nucleophile added to the proximal allyl carbon. Attempts to raise the diastereoselectivity of this reaction through adjusting temperature, solvent or reaction time failed to significantly improve it.

When the reaction sequence to generate the anisole addition product **9D** was repeated using the deuterated benzene complex **1**-_*d6*_, the two protons incorporated in the double protonation sequence were identified by two signals appearing for **6D**-*d_6_* at 3.50 and 1.22 ppm (CD_3_CN), corresponding to the 4-endo and 5-exo positions. These observations reveal two different mechanisms for protonation: consistent with our earlier studies of monoprotonated benzene, the first protonation occurs *syn* to the metal (H_4endo_),^1^ while the second protonation occurs *anti*. The NMR spectrum of **6D**-*d_6_* indicates that even at high acid concentrations, both protonations are highly regio- and stereoselective, with minimal amounts of proton signal (0–15%) appearing at other positions.

**Figure 2:**
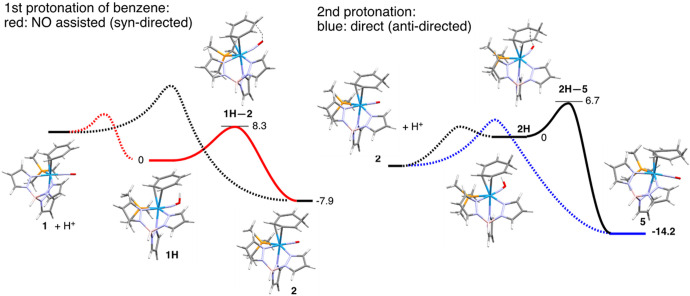
Comparison of first and second protonations of benzene.

DFT calculations support the notion that the first protonation of the benzene ring occurs *syn* to the tungsten via the nitrosonium ligand, where a modest transition state energy of 8.3 kcal/mol is found for proton transfer from the NO oxygen to the ring carbon (red, Fig. 2; SI). Calculations further indicate that an analogous NO-assisted *second* protonation is also viable (~ 7 kcal/mol), provided that protonation of the nitrosonium of **2** can still occur. However, given that the double protonation of **1**-*d_6_* to form **6D**-*d_6_* unambiguously results in a *trans* arrangement of the two ring protons, the second protonation must occur by an intermolecular pathway, anti to the metal. Most likely the comparatively lower transition state energy for direct ring protonation (blue dash in Fig. 2) reflects the considerable thermodynamic preference for **5** over the NO-protonated derivative of **2 (2H**; −14.2 kcal/mol; Fig. 2).

**Figure 3:**
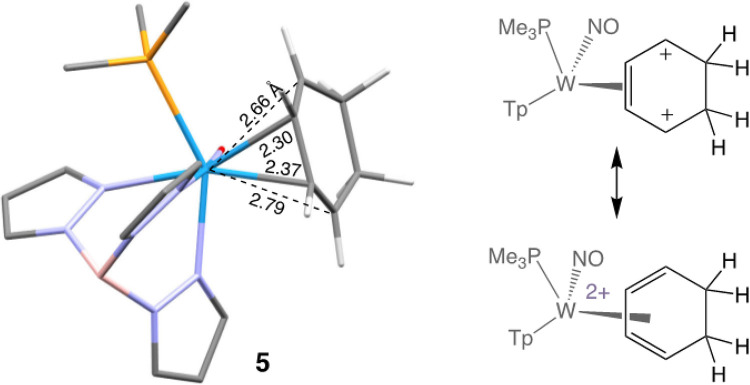
DFT optimized structure of [WTp(NO)(PMe_3_)(C_6_H_8_)]^2+^ (**5**), the result of the double-protonation of the η^2^-benzene ligand of **1**.

According to calculations, the double-protonated benzene complex **5** can be considered as a highly distorted η^4^-tungsten(II)-diene complex (Fig. 3), with elongated bond lengths (2.66, 2.79 Å; *cf*. 2.30, 2.37 Å) between tungsten and the terminal diene carbons. These distorted structural features are reminiscent of those seen for the η^2^-allyl species described earlier. A search of the *Cambridge Structural Database*^8^ failed to identify any analogously distorted η^4^-diene structures; however the structure of **5** is reminiscent to those found in zirconium and hafnium complexes of η^4^-cyclooctatetrene.^9^ The distal carbenium carbon of **5** has the longest bond to the metal (2.79 Å) and might be predicted to be the more reactive site of addition, yet, nucleophilic attack occurs predominantly at the proximal carbenium. Such an addition generates η^2^-allyl species (**6–8D**) with the remaining carbenium distal to PMe_3_. The distal form (**D**) is known to be several kcal/mol more stable than the isomers resulting from distal addition of the arene (**6–8P**).^2^ Hence, we rationalize the kinetic preference for the addition of the arene to the proximal carbenium by invoking a transition state that resembles the product in which the carbenium is distal to the PMe_3_.

The observed 4:1 selectivity discouraged us from developing a synthetic method for enantioenriched cyclohexenes using this approach. Hints of analogous reactivity were observed for the molybdenum complex MoTp(NO)(DMAP)(η^2^-benzene),^10^ (**1-Mo**) including spectroscopic evidence for the Mo analog of **6D** (**6D-Mo**). The large-scale preparation of **1-Mo** and spectroscopic data for **6D-Mo** can be found in the SI. However, the high sensitivity of these compounds to acid ultimately discouraged our further investigation.

**Figure 4:**
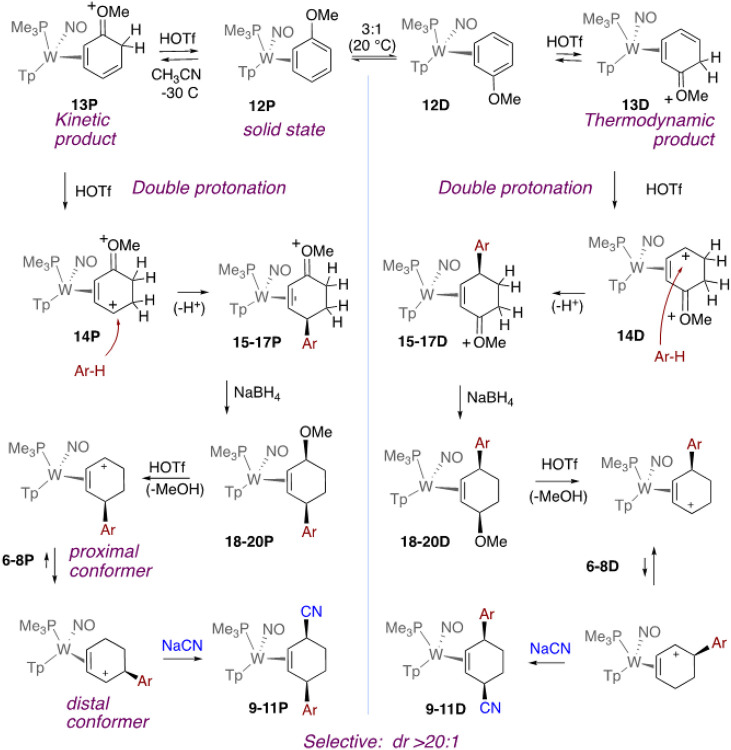
The tungsten-promoted double-protonation of anisole followed by electrophilic aromatic substitution. **6**,**9**,**15**,**18**: Ar-H = anisole; **7**,**10**,**16**,**19**: Ar-H = phenol; **8**,**11**,**17**,**20**: Ar-H = thiophene. Yields for **9**-**11**: 50-70%.

We next considered a modified strategy (Fig. 4) in which an η^2^-anisole complex would be double-protonated. We reasoned that the methoxy substituent would not only facilitate the double protonation, but also could help direct the aryl addition to the *para*- carbon of the anisole, analogous to what we have previously observed for anilines.^11^ In contrast to our aniline observations, we anticipated that the oxocarbenium could be easily reduced later in the reaction sequence. The tungsten anisole complex **12D** exists in solution as a 3:1 equilibrium with its stereoisomer **12P**.^12, 13^ However, the 2H-anisolium complex **13D** has been shown to be thermodynamically favored over its proximal analog **13P** (>20 : 1), again favoring the oxocarbenium carbon in the distal position.^13^ When **13D** was subjected to highly acidic conditions (HOTf/acetonitrile), protonation occurred exclusively at the homoallylic carbon to form the dication **14D** (Fig. 4). Treating this species with the phenol, anisole, and thiophene series resulted exclusively in the enonium species **15–17D**.

Although the anisole complex exists in solution as two interconverting diastereomers (**12D**, **12P**), only **12P** is present in the crystalline solid (prepared from solution precipitation).^14^ Adding a powder of **12P** to a *cold solution* of DPhAT and acetone results exclusively (dr > 20 : 1 P/D) in the 2H-anisolium complex **13P**. This compound, like its benzene analogue, can be protonated a second time to form the dicationic complex **14P**, which can then be elaborated into the η^2^-enonium complexes **15** -**17P** through the additions of anisole, phenol, or thiophene. Subsequent reduction of the η^2^-enonium complexes (**15**–**17**) for either the P or D series generates the allyl ether complexes **18**–**20**, and this is followed by acid-induced loss of methanol to form the π-allyl complexes **6**–**8**. Finally, treatment with NaCN produces the *cis*-3,6-substituted cyclohexene complexes **9**–**11**, but in this case, *each diastereomer can be generated completely free of the complementary diastereomer* (dr > 20 : 1; Fig. 5). We note that even though **6–8P** favor a conformation in which the carbenium is distal to the PMe_3_, steric factors apparently favor addition out of the proximal form (Fig. 4). The cyclohexene product is then liberated with the oxidant NOPF_6_ to generate the organic compounds 4'-hydroxy-1,2,3,4-tetrahydro-[1,1'-biphenyl]-4-carbonitrile (**21**; 70%), 4'-methoxy-1,2,3,4-tetrahydro-[1,1'-biphenyl]-4-carbonitrile (**22**; 34%), and 4-(thiophen-2-yl)cyclohex-2-ene-1-carbonitrile (**23**; 56%). Although beyond the scope of this preliminary study, we note that the arene complexes **1** and **12** can be prepared in enantioenriched form.^2, 15, 16^ Therefore, while this preliminary report only describes racemic mixtures, either hand of the organic *cis*-3,6-cyclohexene would be available from an enantioenriched anisole complex.

In the reaction sequences outlined in Fig. 4, a single regio- and stereoisomer of a *cis*-3,6-disubstituted cyclohexene complex is obtained (**9**–**11**). The synthesis of 3,6-disubstituted cyclohexenes such as **21**–**23** (derived from **9**–**11**) have not been reported previously, despite their relatively simple structures. The closest comparisons are 1,4-dihydronapthalene analogs prepared from a Diels-Alder reaction with benzyne, ^17^ or reaction sequences involving the coupling of aryl halides to cyclohexenes or cyclohexanones. More generally, methods employed to couple aromatics to cycloalkanes typically involve cross-coupling reactions such as Negishi,^18^ Stille,^19^ Suzuki,^20^ and Hiyama couplings,^21^ but such reactions are more difficult than sp^2^-sp^2^ coupling protocols and are often plagued by elimination byproducts. Furthermore, these reactions typically require the use of precious metal catalysts and aryl halides or other suitable aryl precursors. Corey-House,^22, 23^ and Kochi-Schlosser type couplings avoid precious metals but require aryl Grignard reagents.^24^ For cases where an organic arene is utilized, strong Lewis Acid activators are typically required (Friedel-Crafts).^25^ Lewis and Bronsted acids have successfully been used in Friedel-Crafts *alkylations*,^25^ especially in the case of benzyl electrophiles, where rearrangements of the carbocation intermediate are less of an issue. However, examples carried out with high stereoselectivity are rare due to the fact that the electrophile typically passes through a planar sp^2^ intermediate.^25^ The closest comparisons of EAS reactions related to the current study involve cyclohexadienyliumiron complexes combining with anilines or phenols to generate carbazoles.^26, 27^ In these studies, the iron complex does not control the stereochemistry of the reaction and cyclohexadienes or arenes are produced. Limited examples of EAS reactions have also appeared in our own work, in the synthesis of γ-substituted enones.^28^ and tetrahydroindolines.^29^ However, in no case previously were we able to couple these reactions to a second nucleophilic addition. The full scope of cis-3,6-disubstituted cyclohexenes available by this new method, including enantioenriched variations, will be disclosed in due course.

## Figures and Tables

**Figure 5:**
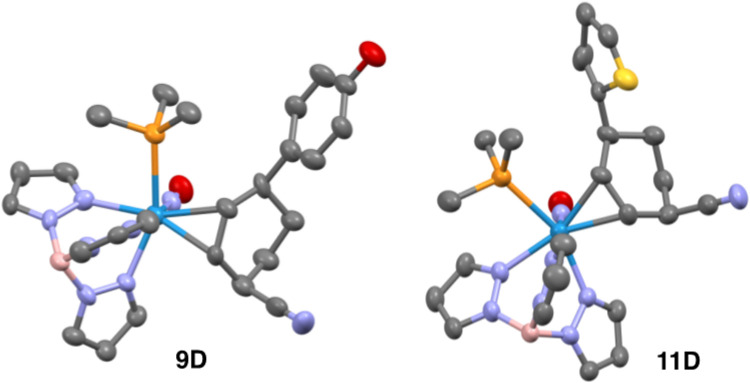
Molecular structure determinations (50% ellipsoids) for **9D** and **11D**.
